# Identification and validation of QTL for grain yield and plant water status under contrasting water treatments in fall-sown spring wheats

**DOI:** 10.1007/s00122-018-3111-9

**Published:** 2018-05-16

**Authors:** Junli Zhang, Shiferaw Abate Gizaw, Eligio Bossolini, Joshua Hegarty, Tyson Howell, Arron H. Carter, Eduard Akhunov, Jorge Dubcovsky

**Affiliations:** 1 Department of Plant Sciences, University of California, Davis, CA 95616 USA; 2 Department of Crop and Soil Sciences, Washington State University, Pullman, WA 99164 USA; 3 Department of Plant Pathology, Kansas State University, Manhattan, KS 66502 USA; 4 Howard Hughes Medical Institute, Chevy Chase, MD 20815 USA

## Abstract

***Key message*:**

**Chromosome regions affecting grain yield, grain yield components and plant water status were identified and validated in fall-sown spring wheats grown under full and limited irrigation.**

**Abstract:**

Increases in wheat production are required to feed a growing human population. To understand the genetic basis of grain yield in fall-sown spring wheats, we performed a genome-wide association study (GWAS) including 262 photoperiod-insensitive spring wheat accessions grown under full and limited irrigation treatments. Analysis of molecular variance showed that 4.1% of the total variation in the panel was partitioned among accessions originally developed under fall-sowing or spring-sowing conditions, 11.7% among breeding programs within sowing times and 84.2% among accessions within breeding programs. We first identified QTL for grain yield, yield components and plant water status that were significant in at least three environments in the GWAS, and then selected those that were also significant in at least two environments in a panel of eight biparental mapping populations. We identified and validated 14 QTL for grain yield, 15 for number of spikelets per spike, one for kernel number per spike, 11 for kernel weight and 9 for water status, which were not associated with differences in plant height or heading date. We detected significant correlations among traits and colocated QTL that were consistent with those correlations. Among those, grain yield and plant water status were negatively correlated in all environments, and six QTL for these traits were colocated or tightly linked (< 1 cM). QTL identified and validated in this study provide useful information for the improvement of fall-sown spring wheats under full and limited irrigation.

**Electronic supplementary material:**

The online version of this article (10.1007/s00122-018-3111-9) contains supplementary material, which is available to authorized users.

## Introduction

Wheat (*Triticum aestivum* L.) is an important crop for global food security that provides roughly one-fifth of the daily calories and dietary proteins consumed by the human population (FAOSTAT 2015). Although more than 700 million metric tons of wheat are produced every year, further increases are required to feed a rapidly growing human population. These increases need to be achieved in environments with changing climatic conditions and with increasing limitations in water and agricultural land (Shiferaw et al. 2013; Curtis and Halford 2014). A better understanding of the genetic basis of yield and yield components under limited irrigation conditions will be required for securing the necessary increases in wheat production.

Water limitations can occur at different times of the wheat growing cycle, and tolerance to these limitations may be governed by different genes depending on the timing or intensity of the water stress. Therefore, a narrow definition of water stress is required to identify genes affecting plant responses to this stress. In this study, water stress was imposed to fall-sown spring wheats by limiting irrigation after the booting stage. QTL affecting grain yield, yield components and plant water status under this condition were compared with QTL obtained under full irrigation.

In many Mediterranean regions, spring wheats are sown in the fall to take advantage of the winter rains. However, winter rains in these regions typically stop prior to the end of the wheat growing season, requiring additional irrigations to maximize grain yield. The identification of genes that positively contribute to grain yield under terminal drought stress can accelerate the development of varieties adapted to this condition and save valuable water resources by reducing the need for terminal irrigation.

Plants can adjust morphologically, physiologically and biochemically to drought stress. Some of these adaptations, for example stomata closing, have a negative impact on growth and grain yield. By contrast, the development of deeper root systems may help plants access water for longer dry periods, maintain water potential and open stomata, and limit yield losses under reduced irrigation (Loutfy et al. 2012). Unfortunately, differences among genotypes in root architecture associated with drought tolerance are difficult to study. It is easier and cheaper to measure the physiological status of the aerial part of the plant and from this information infer the ability of the plant to access water and nutrient resources.

Spectral reflectance indexes (SRI) provide high-throughput nondestructive measures from which several physiological traits can be inferred, including total biomass, plant pigments, canopy photosynthetic capacity, and plant water and nitrogen status (Araus et al. 2002; Babar et al. 2006a, b; Prasad et al. 2007a, b; Bowman et al. 2015). In particular, water indexes have been used successfully to track changes in relative water content, leaf water potential, stomatal conductance and foliage-air temperature differences under water stresses (Peñuelas et al. 1993). Among the different normalized water indexes (NWI) published so far, NWI3 ((R_970_ − R_880_)/(R_970_ + R_880_)) was proposed to be a better predictor of grain yield (Gutierrez et al. 2010) and was selected for this study. This formula is referred to as NWI4 in Prasad et al. 2007a.

To understand better the relationship between water status and grain yield, we measured multiple yield components in both treatments. Yield components often show higher heritability than overall grain yield, providing increased statistical power to detect and map the underlying genes in the different treatments.

Numerous studies have been conducted to identify wheat chromosome regions associated with grain yield components and drought-related traits. Multiple QTL have been identified in all wheat chromosomes and results have been summarized in a meta-QTL analysis for drought and heat stress (Acuña-Galindo et al. 2015) and in a recent review (Gupta et al. 2017).

Most of the initial QTL studies were performed using biparental mapping populations, but genome-wide association studies (GWAS) have become common in recent years (Breseghello and Sorrells 2006; Wang et al. 2012; Edae et al. 2014; Ain et al. 2015; Sukumaran et al. 2015; Zanke et al. 2015). Compared to biparental mapping populations, GWAS populations can be developed faster and provide access to a wider range of alleles (Zhu et al. 2008). However, GWAS can exhibit higher rates of false positives than biparental populations (Yu and Buckler 2006). In young polyploid inbreeding species such as wheat, where linkage disequilibrium (LD) extends over long distances (Chao et al. 2010), GWAS can have limited resolution. By contrast, large biparental populations can generate high resolution genetic maps and have been used effectively in wheat to map-based clone several genes (Uauy et al. 2006; Fu et al. 2009; Zhang et al. 2017). Approaches that combine GWAS and biparental populations (e.g., nested association-mapping populations, NAM) (Yu et al. 2008) can bring together the best of both methods.

In the present study, we conducted GWAS to identify QTL for grain yield, yield components and plant water status in fall-sown spring wheats and focus on those validated in biparental populations. These studies were performed under full and limited irrigation to identify useful QTL for irrigated wheat breeding programs that are interested in saving water by eliminating terminal irrigations.

## Materials and methods

### Plant materials

A collection of 262 common wheat spring lines from several breeding programs (Table S1) was used in the present study. This panel included only photoperiod-insensitive spring varieties, which carry mutations in the *PPD1* homeologs that result in accelerated flowering under short days. Photoperiod-sensitive varieties were excluded because they flower too late in the regions used in this study. This spring wheat panel included 78 genotypes from CIMMYT (CMT), 45 from the University of California, Davis (UCD), 27 from the University of Idaho (UIA), 19 from University of Minnesota (UMN), 15 from Washington State University (WAS), 13 from South Dakota State University (SDK), and 65 from various locations (Table S1). Locations with few cultivars and landraces were grouped together as “Other” in the structure analysis. This group includes accessions from other US programs, and 14 other countries (Table S1).

Eight biparental populations, each including 75 recombinant inbred lines, were used for QTL validation. These populations were generated from crosses between the CIMMYT line Berkut (Irene/Babax//Pastor) as a female parent and eight lines with diverse genetic backgrounds as male parents (three of them included in the GWAS panel). These biparental populations were used only to validate the significant SNPs detected in the GWAS.

Four populations were grown in Davis in 2014 and 2015 under terminal drought: Berkut × PBW343 (population PBW343), Berkut × Dharwar_Dry (population DD), Berkut × LR23 (PI 70613, population LR23), and Berkut × LR3 (CItr 7635, population LR3). The other four biparental populations were grown in Davis, CA and Imperial Valley, CA (2015) under both full irrigation and terminal drought: Berkut × RAC875 (population RAC875), Berkut × Kern _(*Yr5*+*Yr15*+*2NS*+*GPC*-*B1*)_ (population UC1036), Berkut × RSI5 _(*Yr5*+*Yr15*+*Glu*-*A1a*+*GPC*-*B1*)_ (population RSI5), Berkut × Patwin-515HP (PVP 201600390, population UC1419).

### Experimental design

Table S2 summarizes the locations and dates where these populations were sown and harvested, soils, irrigation, fertilization and fungicide treatments, plot sizes, seed densities, and number of blocks used in each experiment. GWAS were conducted in 2 years (2013, 2014) at 3 locations (UC Experimental Field Station in Davis, hereafter, Davis; UC Desert Research and Extension Center in El Centro, CA, located in the Imperial Valley, hereafter, Imperial; and CIMMYT’s Obregon Experimental Station in Yaqui Valley, Sonora, Mexico, hereafter, Obregon, Table S2). GWAS in Imperial and Obregon were each replicated 2 years resulting in five environments: Davis 2014 (Dav14), Imperial 2013 (Imp13), Imperial 2014 (Imp14), Obregon 2013 (Obr13) and Obregon 2014 (Obr14).

Due to the large number of genotypes and limited resources, a non-replicated augmented design (Federer 1956) was used in all the GWAS and biparental population trials. For the GWAS, we used different numbers of blocks in the different experiments, which are detailed in Table S2. Six checks were replicated in all blocks and included Berkut, Blanca Fuerte, Hahn-1RS, Hahn-1RSMA, Patwin-515 and UC1679 in the 2013 experiments. In 2014, UC1679 was replaced by AC Andrew (Table S1). Hahn-1RSMA is an isogenic line of Hahn-1RS with two small 1BS introgressions in the 1RS arm (1RS^ww^) associated with increased susceptibility to drought (Howell et al. 2014). These two lines were included as controls of the water stress response.

In each environment, the complete association panel was grown under two treatments. In the full irrigation treatment, plants received the standard irrigation schedule adjusted by soil moisture. In the terminal drought treatment, plants did not receive irrigation after the booting stage and were under water stress at the grain filling stages. The differences in the number of irrigations between the two treatments were one in Davis 2014 (starting April 23), two in Imperial 2013 (starting April 3) and 2014 (starting March 19), two in Obregon 2013 (starting February 22), and three in Obregon 2014 (starting February 7).

### Trait measurement

Canopy spectral reflectance (CSR) measurements were taken at mid-grain filling stage with spectrometers positioned 50 cm above the canopy. In Imperial 2013, CSR data were collected using an Ocean Optics Jaz spectrometer (www.oceanoptics.com) as described by Howell et al. (2014). In Imperial 2014 and Davis 2014, CSR data were collected using an ASD FieldSpec HandHeld2 portable spectrometer (ASD Inc. Boulder, CO, USA), and the data for each plot were the average of 50 measurements. In Obregon 2013 and 2014, CSR was measured using the CROPSCAN (CROPSCAN Inc. Rochester, MN, USA), GreenSeeker (Trimble Inc. Sunnyvale, CA, USA) and Jaz spectrometers using three to five point measurements per plot. The normalized water index 3 [NWI3 = (R970 − R880)/(R970 + R880)] was used to estimate canopy water status (Gutierrez et al. 2010).

Grain yield (GY, kg/ha) was determined from the grain weight of each plot after harvesting with a Wintersteiger Classic small plot combine (Wintersteiger Inc., Salt Lake City, UT). Plot sizes used in the different experiments are summarized in Table S2. Heading date (HD) was recorded as days from January 1 to the date when 50% of the spikes were fully visible above the flag leaf. Plant height (HT) was determined after maturity as the height of the stem to the tip of the spike excluding awns. QTL from HD and HT are reported only in the supplementary files since they were not the target of this study. QTL for grain yield, yield components and NWI3 that were in significant LD with heading or height QTL were assigned a lower priority for further analyses. Kernel weight (KW) was measured as the average weight of 200 or 400 kernels. Averages for the number of spikelets per spike (SNS) and the kernels per spike (KNS) were estimated from six spikes randomly chosen from each plot.

### Statistical analysis

Data in each environment were adjusted for field variation using a mixed linear model implemented in the R packages “lmerTest” and “lme4” (Bates et al. 2015; Kuznetsova et al. 2017; R Core Team 2017). The mixed model augmented design with un-replicated entries and blocks as random factors is described in the website http://articles.extension.org/pages/60430/introduction-to-the-augmented-experimental-design-webinar (accessed on 01/28/2018). The statistical model used is:

$$ Y_{ij} = \mu + \beta_{i} + \tau_{j} + \varepsilon_{ij} $$

where *β* and *τ* are the effects of blocks and entries, respectively. We used the SAS program AucDes (http://pbgworks.org/sites/pbgworks.org/files/SASprogramAugDes.pdf, accessed on 01/28/2018) for the augmented design. The best linear unbiased predictors (BLUPs) of traits in each environment were obtained and were used for Pearson’s correlation analyses, association mapping, and broad sense heritability estimates. Broad sense heritability (*H*^2^) was estimated using BLUPs and the formula

$$ H^{2} = \sigma_{\text{G}}^{2} /\left( {\sigma_{\text{G}}^{2} + \sigma_{\text{e}}^{2} /r} \right), $$

where *σ*_G_^2^ is the genotype variance, *σ*_e_^2^ is the residual variance, and *r* is the number of environments. Since BLUPs calculated for the augmented designs have no replication within environments, the genotype by environment variance was used as error variance (*σ*_e_^2^ = *σ*_G*E_^2^).

### SNP genotyping

Genotyping was performed at the USDA-ARS genotyping laboratory, Fargo, ND using the Infinium wheat SNP 90K iSelect assay (Illumina Inc., San Diego, CA, USA) developed by the International Wheat SNP Consortium (Wang et al. 2014). This assay yielded 34,138 SNPs, but only 22,226 were used for association-mapping analysis after eliminating those with a minor allele frequency (MAF) < 0.05 (i.e., minor allele present in less than 13 accessions) and/or > 10% missing values. SNP filtering was carried out using Tassel v4.0 (Bradbury et al. 2007). Vernalization genes *VRN*-*A1*, *VRN*-*B1* and *VRN*-*D1* were genotyped using markers described in previous studies (Yan et al. 2004; Fu et al. 2005; Zhang et al. 2008).

The rescaled genetic map from Wang et al. (2014) was used to indicate map locations of these SNPs. The Tagger function of Haploview v4.2 (Barrett et al. 2005) was used to select informative SNPs. To infer population structure, we used 1090 highly informative, non-redundant representative SNPs (tagSNPs) selected using the tagger function *r*^2^ = 0.25, and for the calculation of the kinship matrix, we used 5563 non-redundant SNPs using the tagger function *r*^2^ = 1.0.

Genetic diversity (*D*) of each subpopulation was calculated with the same 5563 SNPs using the formula:

$$ D = 1 - \frac{1}{L}\sum\limits_{l} {\sum\limits_{i} {p_{i}^{2} } } $$

where *p* is the frequency of the *i* allele at the *l* locus and *L* is the number of loci (Weir 1996). D was calculated using SNPs with less than 10% missing values. Calculations were done using different minor allele difference levels (MAFs > 0.1, > 0.05, nd > 0) to facilitate comparisons with previous studies.

Population structure was estimated using STRUCTURE 2.3.4 (Pritchard et al. 2000), as described by Maccaferri et al. (2015). Four hypothetical subpopulations were determined as most likely proxies of the population structure, and the corresponding Q-matrices (4 × 262) of population membership coefficients were obtained. The STRUCTURE plot was drawn using a modified version of the R script STRUCTURE PLOT (Ramasamy et al. 2014). Population structure was also explored by principal component analysis (PCA) using the R package “ade4” (Dray and Dufour 2007).

To quantify the genetic variation explained by sowing times at the places where the lines were originated, we performed an analysis of molecular variance (AMOVA) using the R package “pegas” with 1000 permutations and Euclidean distance as genetic distance (Paradis 2010). Lines from CMT, UCD and other Mediterranean climates were classified as developed under fall sowing (DuF), whereas those from UIA, WAS, UMN, SDK and other high-latitude regions were classified as developed under spring sowing (DuS). Eighteen lines were excluded from this analysis because we did not have sufficient information (Table S1).

### Linkage disequilibrium

Linkage disequilibrium (LD) between markers on each chromosomes was calculated in Tassel v4.3 (Bradbury et al. 2007). The critical *r*^2^ value, beyond which LD was considered to be due to genetic linkage, was determined by taking the parametric 95th percentile of the square-root-transformed *r*^2^ data of unlinked markers (genetic distance > 50 cM) (Breseghello and Sorrells 2006). The scatter plot of *r*^2^ versus genetic distance (cM) was fitted using a nonlinear model described by Remington et al. (2001) in R (R Core Team 2017). This model estimates LD (*r*^2^) using recombination rate and effective population size, and adjusts for a low level of mutation and sample size (Sved 1971; Hill and Weir 1988; Remington et al. 2001). The R function nls (nonlinear least squares method) was used to fit the model. The intersection of the *r*^2^ threshold and the fitted regression was used to estimate the average extent of LD for the complete genome, the three genomes and the individual chromosomes.

### Genome-wide association study

The adjusted traits and the filtered SNPs were used for GWAS using the compressed mixed linear model approach (Yu et al. 2006; Zhang et al. 2010) carried by Tassel v5 (Bradbury et al. 2007) with the implemented EMMA (Kang et al. 2008) and P3D (Zhang et al. 2010) algorithms to reduce computing time. Heterozygous genotypes were treated as missing values in the analysis. The kinship matrix (K matrix) was calculated in Tassel v.4 using the 5563 non-redundant SNPs, and the population structure matrix (Q matrix) from the STRUCTURE analysis described above. The Q + K mixed linear model (MLM) was used for all traits based on comparisons of different methods as described in Maccaferri et al. (2015).

From the GWAS for each trait, we first selected SNPs that were significant (*P *< 0.05, marker-wise) in at least three of the five environments, with at least one environment with highly significant differences (*P *< 0.01). All the SNPs that satisfied these criteria are presented in File S1. Within this primary subset, we then selected those QTL that were also significant (*P *< 0.05) in at least one of the biparental populations in at least two environments. These SNPs are designated hereafter as “validated SNPs” in File S1. SNPs that passed the primary criteria and were in LD at *r*^2^ > 0.2 were grouped together as one QTL, and the SNP with the most significant effect was selected as the representative SNP of the QTL. QTL for different traits that overlap in any of the markers in their, respectively, LD groups were considered colocated.

The probability of an SNP being significant by chance simultaneously for three GWAS and two biparental population experiments was estimated to be less than 6.25E−08 (0.05 × 0.05 × 0.01 × 0.05 × 0.05). Based on this number, the probability of at least one error in the 22,226 SNPs was estimated as 1 − (1 − 6.25E−08)^22,226^ = 0.0014 (per trait). This formula assumes that all the tests are independent, which is not the case for many linked SNPs. If we consider only the 5563 non-redundant SNPs, the probability of one or more false positives among the validated SNPs is estimated to be less than 0.0003 per trait. In summary, the combination of these five selection criteria results in a very stringent selection criterion for the validated SNPs.

Real QTL that were not segregating in the biparental populations or that were not detected because of the small number of lines used per population could be lost using the above criteria. To avoid this, we selected a second set of SNPs that were not validated in the biparental populations but that were highly significant (*P* < 0.01) in the GWAS in at least four environments (three for KNS and SNS that were not evaluated in Obregon). The probability of an SNP being significant by chance in this subset was estimated to be less than 5.5E−05 (or 5.5E−03 for KNS and SNS, using the 5563 non-redundant SNPs). These QTL, henceforth “highly significant non-validated QTL,” are highlighted in File S1 but are not discussed in detail in this study.

## Results

### Population structure analysis reflected geographic origin

**Fig. 1:**
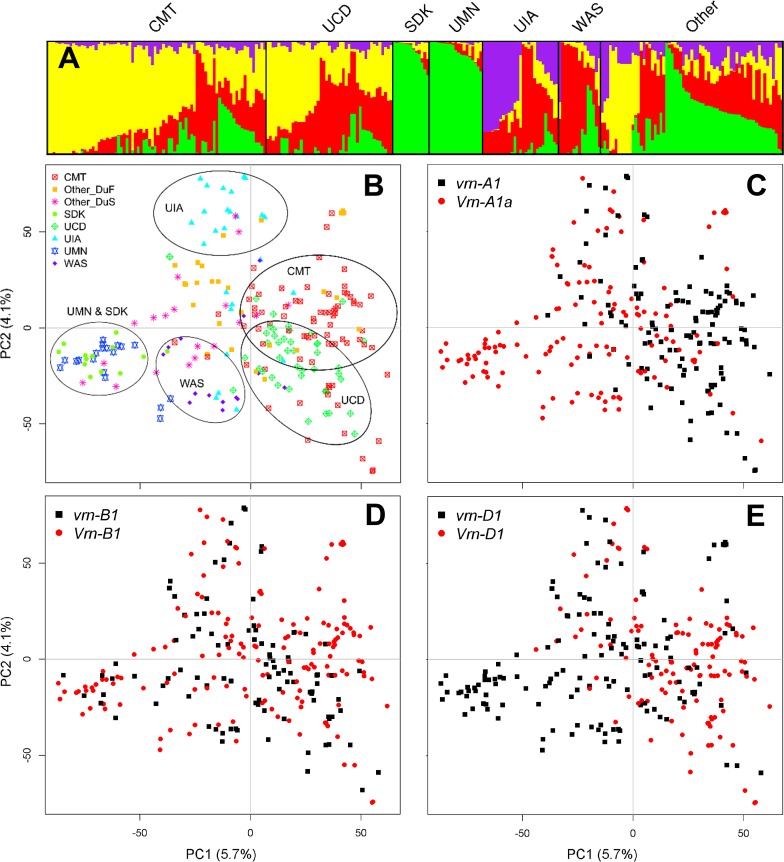
Structure analysis of the 262 lines in the spring wheat association-mapping panel. **a** The STRUCTURE analysis showed four hypothetical subpopulations represented by different colors. First two components (PC1 and PC2) of a principal component analysis of the spring wheat accessions color coded by **b** breeding program and planting time at origin (DuF: developed under fall sowing, DuS: developed under spring sowing), or alleles for the **c** *VRN*-*A1*, **d** *VRN*-*B1* and **e** *VRN*-*D1* genes

Results from STRUCTURE indicated that the 262 wheat lines (Table S1) used in the present study can be grouped into four populations identified by different colors in Fig. 1a, mostly reflecting the geographic origin of the lines. The close relationship between the wheat lines from SDK and UMN is evident in both the STRUCTURE and principal component analyses (PCA, Fig. 1b). The CMT and UCD lines overlap in the first principal component (PC1) and are partially separated by the second principal component (PC2). A slight differentiation between these programs is also evident in the STRUCTURE analysis, where CMT lines showed a higher proportion of the population identified in yellow, whereas UCD showed a higher proportion of the population identified in red. Accessions from UIA and WAS were in the same region of PC1 and were differentiated only by PC2 (Fig. 1b). In the STRUCTURE analysis, genotypes from UIA were mainly a mixture of the populations identified by the red and purple colors, whereas genotypes from WAS were mainly from the population identified in red (Fig. 1a). The group classified as “Other” was a complex mixture of populations, in agreement with their multiple sources (Fig. 1a).

We then classified the accessions into two subgroups based on sowing time at the regions where they were developed (Table S1). The first subgroup included 151 lines developed under fall-sowing (DuF) conditions (UCD, CMT, and OTHER accessions from Mediterranean regions). The second subgroup included 93 lines developed under spring sowing (DuS) conditions (SDK, UMN, UIA, WAS, and OTHER accessions from high latitudes). Eighteen lines were not classified (Table S1). The analysis of molecular variance (AMOVA) showed that sowing time at the place of origin accounted for 4.1% of the total molecular variation, breeding programs within sowing times accounted for 11.7% of the variation, and accessions within breeding programs accounted for the remaining 84.2% (Table 1).

**Table 1:** Analysis of molecular variance (AMOVA) of 220^a^ photoperiod-insensitive spring wheat lines grouped by planting time at the region of origin (fall vs. spring) and by breeding program

Source of variation	DF	Mean square	Variance components	% variation
Among planting time at origin (fall vs. spring)	1	37,518.9	161.9	4.1
Among breeding program within planting time	10	9869.4	466.5***	11.7
Among individuals within breeding program	208	3352.5	3352.5	84.2
Total	219^a^	3806.1	3980.9	

***P < 0.001

^a^This analysis excluded 42 accessions with unknown breeding program in Table S1 (NSG or UNK)

ANOVAs between DuS and DuF accessions, using environments as blocks, showed that DuS accessions headed earlier (3.25 d, *P *= 0.0015), were taller (6.31 cm, *P *< 0.0001) and had lower grain yields (436.7 kg/ha, *P *= 0.0002) than DuF accessions. This last result is not surprising, given that DuF accessions were developed in environments more similar to the ones used in this study than DuS accessions. The reduced grain yield of DuS accessions was associated with decreases in both grain weight (0.5 mg, *P *= 0.025) and grain number (1.2 grains, *P *= 0.027), as well as higher levels of water stress (NWI3, *P *= 0.0007). No significant differences were detected in the number of spikelets per spike between DuS and DuF accessions (*P *= 0.112).

The separation of the DuF and DuS accessions along PC1 (Fig. 1b) correlated well with the distribution of the *VRN*-*A1* and *VRN*-*D1* alleles (Fig. 1c, e). To search for additional loci associated with the differentiation between DuF and DuS accessions, we calculated the fixation index *F*_st_ for individual loci (SNPs with tag 1.0 and representative SNPs for QTL) using the BayeScan program (Foll and Gaggiotti 2008). We detected 286 loci with *F*_st_ values above the 95th percentile generated by bootstrap resampling (10,000 times), which all showed enrichment in opposite alleles in the DuF and DuS accessions (Table S3). When significant loci located at less than 1 cM from each other were combined, the number was reduced from 286 to 112 (Table S3).

### Linkage disequilibrium differed among genomes

The extent of LD in this association panel was estimated based on pairwise LD squared correlation coefficients (*r*^2^) for all intra-chromosomal SNP loci (Fig. S1 and Table S4). The average intra-chromosomal LD in the whole genome was approximately 2.0 cM, similar to the values reported for a diverse panel of 875 spring wheat cultivars and landraces from the core collection at the National Small Grains Collection (NSGC, Maccaferri et al. 2015). The distribution of LD values across genomes was also similar in both studies. The rate of LD decay to the background level in the A and B genomes was faster resulting in a smaller genetic distance (1.3 and 2.1 cM, respectively) than in the D genome (8.0 cM). In this study, LD extent varied across chromosomes, from 0.55 cM for chromosome 7A to 12.84 cM for chromosome 1D (Table S4). The whole genome average critical *r*^2^ value was 0.21, which is similar to the values reported by Mora et al. (2015) and Edae et al. (2014) in their spring wheat mapping panels. In this study, *r*^2^ values varied from 0.17 for chromosome 3D to 0.27 for chromosome 5D.

### Variation in testing environments affected phenotypic differences

**Fig. 2:**
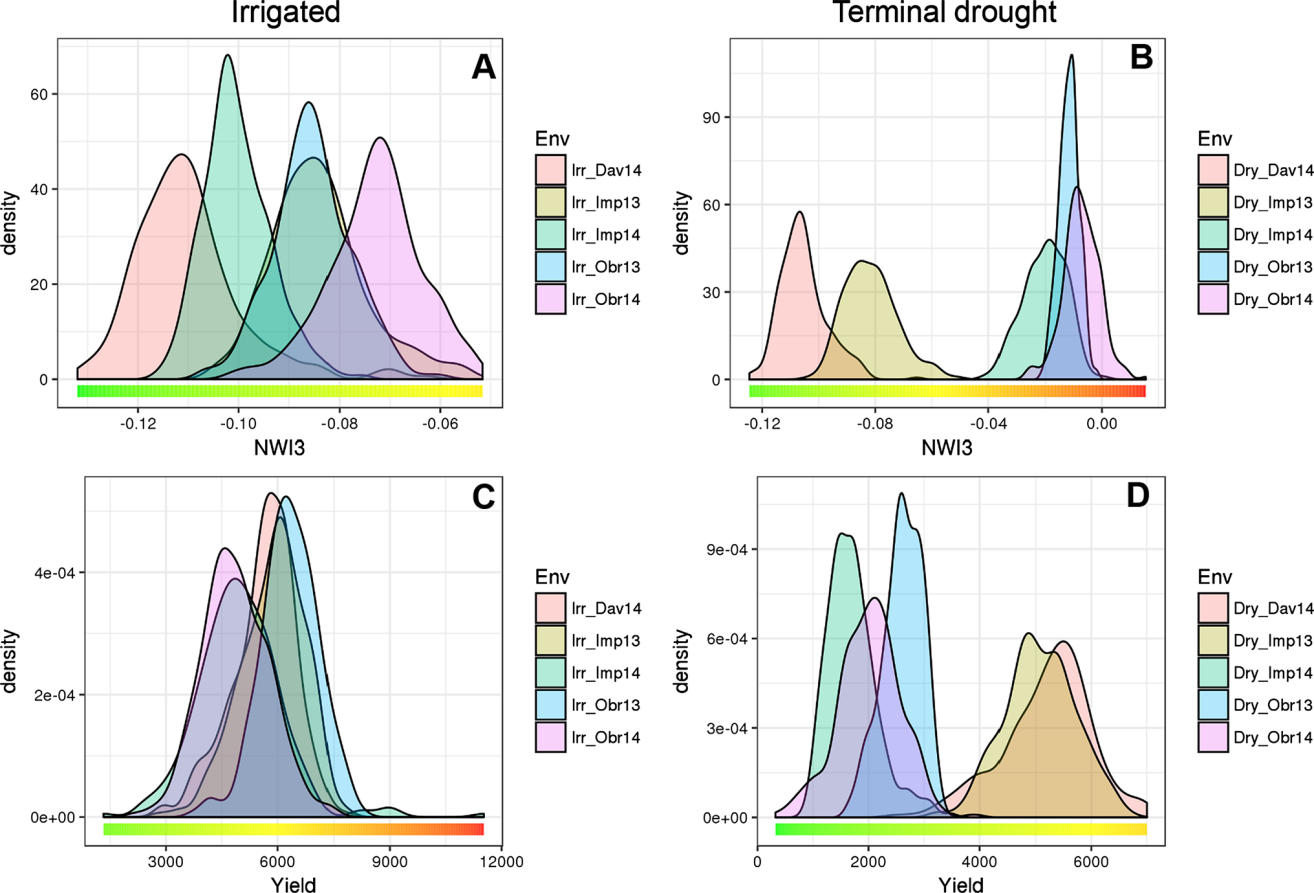
Density plots of **a**–**b** normalized water index 3 (NWI3) and **c**–**d** grain yield (GY). Field experiments performed in five environments under **a**, **c** full irrigation and **b**, **d** terminal drought. NWI3 values that are more positive indicate stronger water stress conditions. Note that the scales for the irrigated and terminal drought conditions are different

Average and range values for yield, yield components and water index in the fully irrigated and terminal drought environments are summarized in Table 2 and Figs. 2 and S2. Out of the five environments, Dav14 and Imp13 were subjected to lower levels of water stress than the other three environments in the terminal drought treatments. In Imp13 (the first experiment in this location), irrigation was stopped late, and in Dav14, natural precipitation in the spring was above average. The lower levels of water stress in these two environments resulted in smaller differences between irrigated and terminal drought treatments for NWI3, grain yield, and plant height relative to those observed in the environments with higher levels of water stress (Table 2, Figs. 2 and S2).

**Table 2:** Averages and standard deviations for seven traits evaluated in the GWAS in five environments under full irrigation (Irr) and terminal drought (Dry) and statistical comparisons

Trait^a^	Env^b^.	Irr		Dry		*P* and *R*^*e*^	Statistical test
		Mean^c^	SD	Mean^c^	SD		
HD	Dav14	93.5a	4.2	93.7a	4.0	0.605	*P* Irr. versus Dry, 5 env
	Imp13*	80.1b	2.6	78.9b	2.6	0.252	*P* Irr. versus Dry, 3 dry env.
	Imp14	74.8c	3.4	74.9c	3.4	− 0.485	Correlation with NWI3
	Obr13*	56.9e	7.5	58.6e	5.1	0.066	P value reg. with NWI3
	Obr14*	63.4d	3.4	63.9d	3.6		
HT	Dav14*	108.4a	9.8	109.5a	10.1	0.156	*P* Irr. versus Dry, 5 env.
	Imp13*	94.7c	9.1	95.7b	8.0	0.121	*P* Irr. versus Dry, 3 dry env.
	Imp14*	91.1d	8	86.2c	10.6	− 0.892	*R* correlation with NWI3
	Obr13*	102.7b	10	72.6d	8.6	0.0005	*P* regression NWI3
	Obr14*	89.1e	6.5	61.6e	4.2		
KW	Dav14	47.4a	4.5	–	–	0.017	*P* Irr. versus Dry, 5 env.
	Imp13*	35.5d	2.5	30.0a	2.8	0.040	*P* Irr. versus Dry, 3 dry env.
	Imp14*	36.0d	2.4	21.9d	2.1	− 0.762	*R* correlation with NWI3
	Obr13*	37.7b	3.7	28.8c	3.2	0.028	*P* regression NWI3
	Obr14*	36.7c	2.7	29.5b	3.2		
GY	Dav14*	5615c	851	5211a	793	0.031	*P* Irr. versus Dry, 5 env.
	Imp13*	5865b	850	5087a	636	0.006	*P* Irr. versus Dry, 3 dry env.
	Imp14*	4985d	1170	1709d	445	− 0.912	*R* correlation with NWI3
	Obr13*	6318a	762	2612b	350	0.0002	*P* regression NWI3
	Obr14*	4873d	893	2021c	554		
KNS	Dav14	46.5c	6.5	46.5b	2.6	0.500	*P* Irr. versus Dry, 5 env.
	Imp13	56.0a	3.5	–	–	0.136	*R* correlation with NWI3
	Imp14*	50.2b	6	48.7a	4.5	0.828	*P* regression NWI3
SNS	Dav14*	21.4b	1.2	18.1b	1.1	0.539	*P* Irr. versus Dry, 5 env.
	Imp13	21.8a	1	–	–	0.187	*R* correlation with NWI3
	Imp14*	20.5c	0.9	20.7a	1.0	0.764	*P* regression NWI3
NWI3^d^	Dav14*	− 0.110d	0.01	− 0.105e	0.01	0.056	*P* Irr. versus Dry, 5 env.
	Imp13*	− 0.084b	0.01	− 0.081d	0.01	0.003	*P* Irr. versus Dry, 3 dry env.
	Imp14*	− 0.100c	0.01	− 0.020c	0.01		
	Obr13*	− 0.085b	0.01	− 0.012b	0.004		
	Obr14*	− 0.073a	0.01	− 0.007a	0.01		

^a^HD, heading date; HT, plant height; KW, kernel weight; KNS, kernels per spike; SNS, spikelet number per spike; GY, grain yield. “–” data not available

^b^Environment with an * showed significant differences between Irr and Dry (*P *<0.05). Dav14 and Imp13 suffered less water stress than the other three environments (referred to as dry environments)

^c^Means with the same letter are not significantly different at *α* = 0.05

^d^More negative values of NWI3 indicate less water stress

^e^Statistical analyses using environment averages as replications. *P* values of comparisons between Irr and Dry (all environments and three under more severe stress). *R* indicate correlations with the NWI3 means and *P* the significance of those correlations

One-way ANOVAs between irrigated and terminal drought environments using environments as blocks revealed significant differences for KW (*P *= 0.017), NWI3 (*P *= 0.056) and GY (*P *= 0.031, Table 2) but not for the other traits (HD, HT, KNS, and SNS). When only the three environments with higher levels of water stress were included in the analysis, the differences in NWI3 and GY between irrigated and terminal drought treatments became more significant (NWI3 *P *= 0.003 and GY *P *= 0.006, Table 2).

### Correlations among traits and broad sense heritability

Using location averages as replications, NWI3 showed significantly negative correlations with GY (*R *= − 0.912, *P *= 0.0002), KW (*R *= − 0.762, *P *= 0.028) and HT (*R *= − 0.892, *P *= 0.0005), and a marginally nonsignificantly negative correlation with HD (*R * = − 0.482, *P *= 0.066, Table 2). As expected, in the environments with stronger water stress plants tended to be shorter, headed earlier, produced lighter grains and yielded less than plants in the environments with lower levels of water stress.

We also performed correlation analyses among traits within locations using the 262 individual accessions (Table 3). In both treatments at all five environments, NWI3 was negatively correlated with HD, yield, KW (except Imp14), KNS, and SNS (Table 3). The correlations between NWI3 and HT were more variable, with positive correlations in some environments and negative in others. However, the directions of the correlations were consistent between fully irrigated and terminal drought treatments within each environment.

**Table 3:** Correlation among NWI3, grain yield (GY), grain yield components, heading date and plant height in each environment under full irrigation (Irr) and terminal drought (Dry)

Trait^a^	Irr					Dry				
	Dav14	Imp13	Imp14	Obr13	Obr14	Dav14	Imp13	Imp14	Obr13	Obr14
NWI3_vs_GY	− 0.61***	− 0.42***	− 0.38***	− 0.45***	− 0.52***	− 0.52***	− 0.56***	− 0.11	− 0.28***	− 0.46***
NWI3_vs_HD	− 0.40***	− 0.48***	− 0.45***	− 0.33***	− 0.12*	− 0.34***	− 0.24***	− 0.54***	− 0.06	− 0.05
NWI3_vs_HT	− 0.01	0.1	0.25***	− 0.24***	− 0.18**	− 0.11	0.18**	0.48***	− 0.14*	− 0.28***
NWI3_vs_KW	− 0.13*	− 0.14*	0.14*	− 0.15*	− 0.21***	–	− 0.40***	− 0.16*	− 0.24***	− 0.12
NWI3_vs_KNS	− 0.27***	− 0.33***	− 0.28***	–	–	− 0.30***	–	− 0.1	–	–
NWI3_vs_SNS	− 0.22***	− 0.25***	− 0.31***	–	–	− 0.31***	–	− 0.25***	–	–
GY_vs_HD	0.59***	0.08	0.15*	0.27***	− 0.08	0.48***	− 0.06	− 0.22***	− 0.43***	− 0.24***
GY_vs_HT	− 0.16**	− 0.35***	− 0.31***	0.07	0.14*	− 0.18**	− 0.17**	0.12*	0.55***	0.43***
GY_vs_KW	0.09	0.29***	0.25***	0.23***	0.35***	–	0.47***	0.64***	0.25***	0.40***
GY_vs_KNS	0.58***	0.33***	0.22***	–	–	0.44***	–	0.21***	–	–
GY_vs_SNS	0.37***	0.06	0.15*	–	–	0.39***	–	− 0.06	–	–
HD_vs_HT	− 0.14*	− 0.36***	− 0.29***	− 0.20**	− 0.31***	− 0.13*	− 0.37***	− 0.52***	− 0.58***	− 0.31***
HD_vs_KW	− 0.13*	− 0.17**	− 0.37***	− 0.27***	− 0.32***	–	− 0.24***	− 0.21***	− 0.04	− 0.29***
HD_vs_KNS	0.49***	0.19**	0.22***	–	–	0.35***	–	0.08	–	–
HD_vs_SNS	0.28***	0.39***	0.24***	–	–	0.35***	–	0.30***	–	–
HT_vs_KW	0.04	0.11	0.08	0.31***	0.25***	–	0.23***	0.28***	0.22***	0.27***
HT_vs_KNS	0.04	− 0.15*	− 0.20**	–	–	0.08	–	− 0.20***	–	–
HT_vs_SNS	− 0.14*	− 0.18**	− 0.16**	–	–	0.03	–	− 0.18**	–	–
KW_vs_KNS	− 0.24***	− 0.04	− 0.20**	–	–	–	–	0.09	–	–
KW_vs_SNS	− 0.17**	− 0.22***	− 0.20**	–	–	–	–	− 0.1	–	–
KNS_vs_SNS	0.48***	0.41***	0.43***	–	–	0.66***	–	0.28***	–	–

*P < 0.05, **P < 0.01, ***P < 0.001, others are not significant (*P *≥ 0.05) or not available (−)

^a^HD, heading date; HT, plant height; KW, kernel weight; KNS, kernel number per spike; SNS, spikelet number per spike; GY, grain yield; NWI3, normalized water index 3

Grain yield showed positive correlations with HD in the irrigated environments but negative correlations in the terminal drought (except in the limited stressed Dav14 environment, Table 3), suggesting that late flowering plants had an advantage when water was available but a disadvantage when irrigation was interrupted before grain filling. Grain yields showed negative correlations with HT in the environments with low water stress but significantly positive correlations in the three environments under high water stress (Imp14, Obr13 and Obr14, Table 3). This suggests that taller plants performed better in the water-stressed environments. As expected, GY showed positive correlations with KW, KNS and SNS yield components, which were significant in some of the environments.

HD showed consistent negative correlations in both environments with HT and KW. The first result suggests that late heading plants tended to be shorter. This may be related to the overall trend of DuS accessions to be both earlier and taller. The negative correlations between HD and KW were consistent in both irrigations and more likely reflected the negative effect of heat stress on grain development in late flowering plants. Interestingly, all the significant correlations between HD and KNS/SNS were positive suggesting that plants with a more extended development may also have a longer spike development period and more time to add additional spikelets and grains. As expected, KW and KNS were negatively correlated, and KNS and SNS were positively correlated (Table 3). All traits, except HD, showed higher heritability under the full irrigation treatments than under the terminal drought treatments (Table 4). GY and KNS showed similar but lower heritability than the other traits (Table 4).

**Table 4:** Broad sense heritability (*H*^2^) and variance components for the best linear unbiased predictors (BLUPs) of seven traits across the tested environments under full irrigation (Irr) and terminal drought (Dry) conditions

Treatment	Trait	No. env.	σ_G_^2^	σ_E_^2^	σ_e_^2^	H^2^
Irr	HD	5	12.5	205.8	8.3	0.88
	HT	5	53.2	66.4	23.5	0.92
	Yield	5	253,615	365,729	583,496	0.68
	KW	5	6.0	24.5	4.8	0.86
	SNS	3	0.7	0.4	0.4	0.84
	KNS	3	11.1	22.9	19.1	0.64
	NWI3	5	2.3E−05	2.2E−04	5.4E−05	0.68
Dry	HD	5	10.0	187.7	4.7	0.91
	HT	5	48.7	354.4	25.4	0.91
	Yield	5	49,517	2,869,254	282,193	0.47
	KW	4	4.4	14.2	3.7	0.83
	SNS	2	0.4	3.5	0.6	0.59
	KNS	2	3.5	2.5	10.3	0.40
	NWI3	5	6.9E−06	2.0E−03	4.6E−05	0.43

σ_G_^2^  =  genotype variance; σ_E_^2^  =  environment variance; σ_e_^2^   =   residual variance (here   =   σ_GE_^2^ since BLUPs have a single replication per environment); *H*^2^   =   broad sense heritability. All genotype and environment variance were significant at *P* < 0.001

### Multiple QTL were validated in biparental populations

**Fig. 3:**
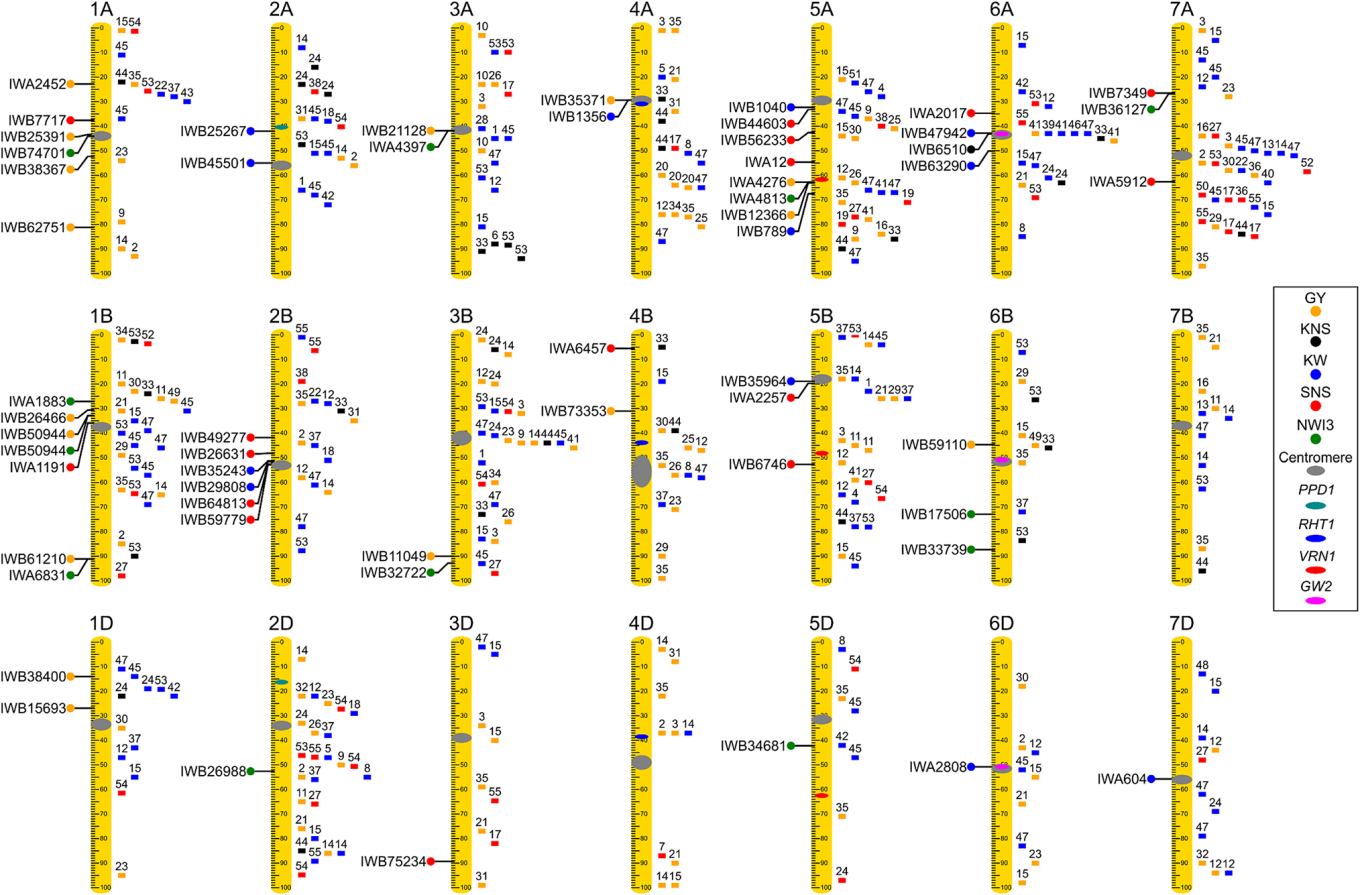
Chromosome location of QTL for water status (NWI3 = green), grain yield (GY = orange), kernel weight (KW = blue), spikelet number per spike (SNS = red), and kernel number per spike (KNS = black). Chromosome genetic lengths were standardized to the same relative length of 100. Validated SNPs identified in this study are indicated to the left of the chromosomes, whereas previously mapped QTL are indicated to the right side of the chromosomes with numbers on top referencing the publications listed in File S2, which also describes the mapping population used and the confidence intervals. Known genes and centromere positions are indicated within the chromosome rulers

All the SNPs that showed significant differences between alleles for any of the traits in at least three environments (at least one highly significant *P *< 0.01) are reported in File S1. Among those, we focused on the SNPs validated in the biparental populations (Table 5), which are described below for each trait. The position of the validated SNPs on standardized chromosomes is presented in Fig. 3, where they are compared with previously published QTL indicated to the right of the chromosomes. The number above the previously mapped QTL refers to File S2, which includes the references and the genetic maps used in the comparison (from GrainGenes https://wheat.pw.usda.gov/GG3/).

**Table 5:** Validated SNPs for QTL for grain yield (GY), normalized water index 3 (NWI3), kernel number per spike (KNS), kernel weight (KW), and spikelet number per spike (SNS), in the spring wheat association-mapping panel, with their genetic position, favorable allele and its average frequency, the *P* values in each environment and the colocated QTL for other traits

Trait	Marker^a^	Chr.	Position^b^	Fav. allele^c^	Frequency	Dav14_Dry	Imp13_Dry	Imp14_Dry	Obr13_Dry	Obr14_Dry	Dav14_Irr	Imp13_Irr	Imp14_Irr	Obr13_Irr	Obr14_Irr	Colocated
GY	IWA2452	1A	36.7	A	0.83	ns	ns	ns	**	**	ns	ns	ns	*	ns	
	IWB25391	1A	70.1	C	0.75	ns	ns	ns	*	ns	ns	ns	ns	**	*	
	IWB38367	1A	84.3	G	0.94	*	ns	ns	ns	ns	ns	ns	*	***	*	
	IWB62751	1A	130.9	G	0.49	ns	ns	**	ns	ns	ns	*	*	ns	ns	
	IWB26466	1B	53.3	C/T	–	ns	ns	**	ns	ns	ns	ns	*	ns	**	
	IWB50944	1B	57.3	A/G	–	ns	ns	***	ns	*	ns	ns	*	ns	*	NWI3
	IWB61210	1B	158.6	G	0.33	*	ns	ns	*	**	ns	ns	ns	ns	ns	
	IWB38400	1D	29.2	C	0.76	ns	**	*	ns	ns	ns	**	ns	ns	ns	HT
	IWB15693	1D	56.3	A	0.16	*	ns	ns	ns	*	*	ns	ns	**	ns	
	IWB21128	3A	86.7	T	0.40	*	ns	ns	ns	ns	**	ns	ns	*	*	NWI3
	IWB11049	3B	140.5	T	0.79	ns	ns	ns	**	**	*	ns	ns	ns	ns	NWI3
	IWB35371	4A	49.0	G	0.35	ns	ns	ns	**	**	ns	ns	ns	ns	*	KW
	IWB73353	4B	38.1	C	0.70	ns	ns	*	ns	ns	ns	**	*	ns	ns	
	IWA4276	5A	93.2	A/G	–	ns	ns	ns	*	ns	ns	**	ns	*	ns	NWI3,HD
	IWB12366	5A	93.8	C	0.47	ns	ns	ns	ns	**	ns	*	ns	*	ns	
	IWB59110	6B	57.0	C/T	–	ns	ns	**	ns	**	ns	ns	*	ns	ns	
NWI3	IWB74701	1A	71.1	G	0.68	*	*	ns	ns	ns	*	ns	**	ns	ns	
	IWA1883	1B	47.2	T	0.36	**	ns	ns	ns	ns	ns	ns	*	ns	*	
	IWB50944	1B	57.3	A	0.22	*	ns	ns	ns	*	ns	ns	ns	ns	**	GY
	IWA6831	1B	158.6	T	0.75	*	ns	**	ns	ns	*	ns	*	ns	ns	HD
	IWB26988	2D	80.4	G/A	–	ns	**	ns	ns	*	ns	ns	*	**	ns	
	IWA4397	3A	86.7	G	0.40	***	ns	ns	ns	ns	*	ns	*	ns	ns	GY
	IWB32722	3B	144.7	T	0.77	*	ns	ns	ns	*	**	ns	ns	ns	ns	GY,HT
	IWA4813	5A	93.2	T	0.55	ns	ns	*	ns	ns	ns	ns	*	**	ns	GY
	IWB34681	5D	94.6	A/G	–	ns	ns	**	ns	*	ns	ns	ns	ns	*	
	IWB17506	6B	93.0	G	0.29	ns	*	ns	ns	ns	*	*	ns	**	ns	
	IWB33739	6B	111.3	A/G	–	ns	ns	**	ns	*	ns	ns	ns	ns	**	
	IWB36127	7A	65.7	T	0.84	*	ns	ns	ns	ns	*	**	*	ns	ns	SNS, HD
KNS	IWB6510	6A^d^	79.1	C	0.86	ns	–	*	–	–	**	*	ns	–	–	
KW	IWB25267	2A	77.9	A	0.61	–	*	ns	ns	ns	*	ns	ns	***	*	
	IWB45501	2A	102.0	C	0.45	–	*	*	ns	ns	**	ns	**	*	*	HT
	IWB35243	2B	97.0	C	0.63	–	*	**	*	*	**	ns	ns	*	*	
	IWB29808	2B	97.3	C	0.28	–	**	**	ns	*	ns	ns	*	ns	*	
	IWB1356	4A	49.0	G	0.47	–	*	ns	**	*	ns	ns	ns	ns	*	GY
	IWB1040	5A	48.0	T	0.52	–	ns	ns	***	*	ns	*	**	*	*	SNS
	IWB789	5A	100.2	T	0.53	–	*	ns	*	ns	**	ns	ns	*	**	
	IWB35964	5B	41.6	T	0.23	–	ns	ns	ns	ns	*	*	*	*	**	SNS
	IWB20366	5B^d^	49.6	G	0.83	–	ns	*	ns	**	ns	*	ns	**	**	
	IWB47942	6A	78.5	G	0.67	–	**	ns	*	ns	***	**	**	*	*	
	IWB63290	6A	91.9	G	0.28	–	***	***	**	**	*	*	*	ns	*	
	IWA2808	6D	82.1	A	0.47	–	ns	ns	*	**	*	*	**	**	***	HD
	IWA604	7D	134.4	G	0.67	–	**	*	*	ns	*	ns	ns	*	ns	
SNS	IWB7717	1A	60.7	A	0.84	*	–	*	–	–	*	***	*	–	–	
	IWA1191	1B	62.6	G	0.27	ns	–	*	–	–	**	ns	*	–	–	
	IWB49277	2B	79.0	A	0.17	ns	–	ns	–	–	*	**	*	–	–	
	IWB26631	2B	91.0	T	0.74	*	–	ns	–	–	ns	*	**	–	–	
	IWB64813	2B	99.2	T	0.60	ns	–	*	–	–	ns	*	**	–	–	
	IWB59779	2B	99.7	C	0.81	**	–	**	–	–	ns	*	ns	–	–	HT
	IWB34446	2B^d^	126.1	T	0.73	**	–	*	–	–	***	**	ns	–	–	
	IWB75234	3D	148.4	A	0.93	ns	–	**	–	–	ns	*	*	–	–	
	IWA6457	4B	6.8	G	0.64	*	–	**	–	–	*	*	ns	–	–	
	IWB44603	5A	49.2	A	0.44	ns	–	*	–	–	*	**	**	–	–	KW
	IWB56233	5A	63.2	G	0.77	**	–	ns	–	–	**	*	*	–	–	
	IWA12	5A	81.1	C	0.58	ns	–	**	–	–	ns	*	*	–	–	
	IWA2257	5B	43.3	T	0.77	*	–	ns	–	–	ns	*	**	–	–	KW
	IWB6746	5B	115.7	T	0.13	ns	–	***	–	–	ns	**	**	–	–	
	IWA2017	6A	63.7	C	0.73	*	–	ns	–	–	**	*	ns	–	–	HD
	IWB7349	7A	64.6	T	0.67	***	–	ns	–	–	**	**	*	–	–	NWI3
	IWA5912	7A	152.8	T	0.81	***	–	***	–	–	***	***	***	–	–	

^a^Only SNPs that passed the two selection criteria in GWAS and biparental populations are presented in this table. The complete SNP list for each QTL that passed only the first criteria is reported in File S2. *ns* not significant, *P < 0.05, **P < 0.01, ***P < 0.001, “–” not available

^b^Chromosomes and positions are based on the 90 K consensus map

^c^The presence of two alleles in this column indicates opposite effects in different environments

^d^Map position determined from a marker on LD

The position of the validated SNPs for HD and HT is presented in File S1 and Fig. S3 for comparative purposes. We found nine HD and HT QTL that were colocated with grain yield, yield components and water status QTL (Table 5), which is consistent with the significant correlations among these traits (Table 3). Given the strong effect of heading time and plant height on environment adaptation, it is likely that the colocated yield QTL are pleiotropic effects of genes affecting HD and HT. Finally, the highly significant non-validated SNPs (*P *<0.01 in at least 4 environments) are highlighted in File S1 and summarized in Fig. S4.

#### QTL for grain yield

Sixteen QTL for grain yield were identified and validated on nine different chromosomes (Table 5). Twelve of these QTL showed consistent effects across environments, but four showed opposite effects in different environments (Table 5). These differences were not correlated with irrigation treatments suggesting that they were associated with other unknown environmental effects. These variable QTL together with QTL 1D_IWB38400_ and 5A_IWA4276_ colocated with HT and HD QTL were placed in a lower priority list for future studies and breeding applications.

Four GY QTL (1B_IWB50944_, 3A_IWB21128_, 3B_IWB11049_, and 5A_IWA4276_) were in LD with SNPs that were validated for NWI3; and two more yield/NWI3 QTL pairs (1A_IWB25391_/1A_IWB74701_ and 1B_IWB61210_/1B_IWA6831_) were less than 1 cM apart but not in LD (Table 5). The colocation of several QTL for grain yield and NWI3 is in agreement with the significantly negative correlation observed among the means of these traits from different environments (Table 2) and among line values within environments (Table 3). The colocation of grain yield QTL 4A_IWB35371_ with a QTL for kernel weight (Table 5) is also consistent with the significantly positive correlation between these two traits (Table 3).

Among the 12 QTL with consistent effects across environments, the favorable allele is the most frequent for half of them (Tables 5 and S5). The average frequency of the favorable allele was generally consistent across breeding programs, with the exceptions of 1A_IWB25391_, 1D_IWB15693_, 3A_IWB21128_, and 5A_IWB12366,_ which showed unusually low frequencies in SDK and UMN (Table S5).

#### QTL for NWI3

Among the 12 QTL for NWI3 identified and validated in this study, nine showed consistent effects across environments (Table 5). Three NWI3 QTL showed opposite effects in different environments (Table 5), and the differences were not correlated with differences in irrigation. These three variable QTL, together with NWI3 QTL 1B_IWA6831_, 3B_IWB32722_, and 7A_IWB36127_ that were colocated with HT and HD QTL, were placed in a lower priority list for future studies.

Even though NWI3 and grain yield showed the lowest heritability among the traits described in Table 4, eight of the twelve NWI3 QTL were colocated or closely linked (< 1 cM) with QTL for other traits, providing indirect support for their validity. Most NWI3 QTL showed balanced frequencies among breeding programs. However, the UMN and SDK programs showed complete absence of the favorable alleles for NWI3 QTL 1B_IWA1883_ and 5A_IWA4813_, and low frequencies (< 10%) 1B_IWB50944_ and 3A_IWA4397_ (Table S5).

#### QTL for kernel weight (KW)

Thirteen KW QTL were identified and validated on eight chromosomes and all showed consistent effects across locations. Two of the KW QTL were associated with HD (6D_IWA2808_) or HT QTL (2A_IWB45501_, Table 5) and may be pleiotropic effects of genes affecting flowering or plant height.

Kernel weight showed high heritability (Table 4), which was reflected in a high proportion of environments that were significant (58%), one-third of which were highly significant (Table 5). Although most of the KW QTL showed significant effects under both terminal drought and full irrigation, QTL 5B_IWB35964_ was significant only in the fully irrigated treatment (Table 5).

Among the 11 KW QTL that were consistent across environments and did not overlap with HD or HT QTL, the favorable allele was the most frequent allele in seven of them (Tables 5 and S5) suggesting the possibility that breeders have been selecting for the favorable alleles. The average frequency of the favorable allele at these 11 loci was similar across breeding programs, except for 2B_IWB29808_ (< 5% in UMN and SDK), 5B_IWB35964_ (< 10% in SDK and UCD and absent in UMN) and 6A_IWB47942_ which was fixed for the favorable allele in WAS (Table S5).

#### QTL for kernel number per spike (KNS)

Only one validated QTL for KNS was detected on the long arm of chromosome 6A, closely linked to but not colocated with the KW QTL 6A_IWB47942_ (Fig. 3). Multiple QTL for KW, KNS and GY have been published before in this chromosome region (Fig. 3) providing additional validation for this QTL. The favorable allele of the KNS QTL was fixed (SDK, UIA, UMN, and WAS) or almost fixed (> 0.93) in most programs but showed a lower frequency at CMT (0.61) and may be of use there (Table S5).

#### QTL for spikelet number per spike (SNS)

We identified and validated 17 QTL for SNS, and all were consistent across environments (this trait was not evaluated at Obregon). SNS QTL 2B_IWB59779_ and 6A_IWA2017_ co-segregated with QTL for plant height and heading date. SNS QTL 5A_IWB44603_ and 5B_IWA2257_ were in LD with a QTL for kernel weight, and in both cases, the alleles for higher number of spikelets per spike were associated with lighter grains. This is an expected result given the negative correlation observed between these two traits (Table 3).

The high heritability of this trait (Table 4) was reflected in a large proportion of significant QTL across environments (Table 5). QTL 7AL_IWA5912_ and 1A_IWB7717_ were significant in all five locations, and another six were significant in four out of the five tested locations (Table 5). QTL 7AL_IWA5912_ was the most significant QTL (*P *<0.001) across multiple environments. The favorable allele for this QTL is present at a high frequency (> 0.75, Tables 5 and S5) across programs.

## Discussion

### Gene diversity in the spring wheat AM panel

The panel of 262 photoperiod-insensitive spring wheat lines used in this study included varieties and lines from CIMMYT and US breeding programs and a small number of landraces and accessions from different parts of the world to provide a good representation of spring wheat genetic diversity. The selected lines originated from regions with Mediterranean climates, where spring wheat varieties are sown in fall (DuF), and from high-latitude regions, where spring wheats are sown in spring (DuS). The genetic diversity of the complete panel (*D* = 0.35, using MAF > 0.05) was similar to the diversity within the DuF (*D* = 0.33) and DuS (*D* = 0.35) subgroups. Even within the individual breeding programs, D varied only between 0.26 and 0.31 (Table S6). These results are consistent with those obtained from the AMOVA, which showed a low proportion of variation between DuS and DuF (4.1%) and among breeding programs (11.3%) relative to the variation detected among accessions within individual breeding programs (84.2%). These data suggest that the breeding programs included in this study have a good representation of the genetic variation available among spring wheat varieties.

We compared the *D* values calculated in this study with those from a previous study including 875 spring wheat accessions from the NSGC (Maccaferri et al. 2015). To make the results of both studies comparable, we recalculated the estimated genetic diversity in our panel using loci with MAF > 0.10. At this MAF threshold, our D value estimate (*D* = 0.38) was close to the one estimated in the NSGC study (*D* = 0.40, Table S6). This result suggests that our panel captured a substantial proportion of the genetic diversity present in the core spring wheat NSGC despite including only photoperiod-insensitive accessions.

Finally, we compared our results with those published by Chao et al. (2010), where the estimates of diversity were calculated using SNPs with at least one polymorphic accession (MAF > 0) and applying the formula from Botstein et al. (1980), which usually results in smaller *D* values. To perform a valid comparison, we estimated our *D* values using loci with MAF > 0 and recalculated the *D* values from Chao et al. (2010) using the D formula from Weir (1996) as done in this study. The recalculated D values were similar in the two studies, both for the overall values (0.28 vs. 0.24, Table S6) and for the values of individual breeding programs. The only differences were slightly higher *D* values for CMT and SDK in this study than in the data recalculated from Chao et al. (2010) (Table S6).

### Differentiation between spring-sown and fall-sown spring wheats

The genetic relationships among breeding programs determined by PCA and STRUCTURE analyses were consistent with their geographical proximity, which likely favors germplasm exchanges. In the PCA, the first principal component separated lines from breeding programs that sow their materials in the fall from those that sow their lines in the spring. Although the number of breeding programs included in this study is too small to determine if planting time in the region of origin is the main factor behind this separation, two independent observations provide indirect support for this hypothesis. First, 65 landraces and breeding lines sampled from different parts of the world were also separated by PC1 when they were classified according to sowing time at their regions of origin (Fig. 1b). Second, PC1 showed a clear gradient for the *VRN*-*A1* and *VRN*-*D1* alleles (Fig. 1c, e), which have been reported before to be correlated with sowing time in other parts of the world (Gotoh 1979; Stelmakh 1990, 1998; Goncharov 1998; Iwaki et al. 2000, 2001).

Wheat varieties planted in the spring need to complete their growth cycle in a short time, which favors the selection for the strong *Vrn*-*A1a* spring allele (Yan et al. 2004). Once *Vrn*-*A1a* is present, the additional presence of *Vrn*-*D1* or *Vrn*-*B1* has limited effect. By contrast, spring wheat varieties sown in the fall benefit from the absence of the strong *Vrn*-*A1a* spring allele and the presence of the milder *Vrn*-*D1* and/or *Vrn*-*B1* alleles, which exhibit a small residual vernalization requirement (Zhang et al. 2008) and determine a longer growing cycle. In our study, 81.7% of the spring-sown accessions carried the strong *Vrn*-*A1a* allele for spring-growth habit and 88.7% of the fall-sown accessions carry the mild *Vrn*-*D1* or *Vrn*-*B1* alleles. Similar patterns were reported in a survey of 150 spring-sown and 68 fall-sown Chinese spring wheat varieties (Zhang et al. 2008).

Since all the field experiments performed in this study were sown in the fall, it is not surprising that DuF accessions developed in a similar environment performed better than DuS accessions that were bred for a shorter growing season. Even though little genetic differentiation was observed between the DuS and DuF accessions (4.1% based on AMOVA), on average, DuS accessions headed earlier were taller and showed reduced grain weight, grain number per spike, and grain yield than DuF accessions. Based on these results, we hypothesize that DuF and DuS accessions may differ from each other in a subset of genes important for adaptation to the different sowing times (e.g., growth cycle length, frost tolerance, disease resistance). This hypothesis is supported by the detection of 286 loci (5.1% of the analyzed loci) with significant *F*_st_ values (Table S3, Fig. S3). Among these loci, we detected five that were also significant and validated in our QTL analysis (IWB12366 for GY, IWB63290 for KW, IWB6510 for KNS, and IWB76814 and IWB78184 for HT).

### QTL validation

The main objective of this study was to find useful and stable alleles to improve yield potential in fall-planted spring wheats. To favor the selection of stable effects, we selected QTL that were significant in multiple environments. This strategy allowed us to use less stringent thresholds for the detection of QTL in the individual environments, while maintaining a stringent selection criterion for the overall analysis. We first used GWAS to interrogate simultaneously a large number of alleles and then biparental populations to validate a subset of the detected QTL. This validation step is necessary to select a parental line confirmed to have the beneficial allele that can be used as donor in the breeding program.

To test if our selection criteria were too stringent, we checked if the validated QTL included genes with known effects on the traits measured in this study. For HT, we detected significant SNPs close to *RHT*-*B1* and *RHT*-*D1* (Peng et al. 1999), which are the main genes affecting wheat plant height (Fig. S3). For GW, we identified QTL 6A_IWB47942_ and 6D_IWA2808_ in the centromeric region, where the *GRAIN WEIGHT 2* homeologs are located (Simmonds et al. 2016). We also found significant QTL 2B_IWB35243_ and 2B_IWB29808_ on chromosome arm 2BS linked to the grain weight gene *TaSUS2*-2B (Jiang et al. 2011) (Fig. 3).

However, several known flowering genes were not included among the validated SNPs for HD. We found many of these genes in the second subset of highly significant non-validated SNPs (*P *<0.01 in four environments but not validated in the biparental populations, File S1 and Fig. S4). In this additional subset, we identified significant HD QTL 1A_IWA1644_ and 2D_IWA989_ closely linked to the earliness per se gene *Elf3* (Alvarez et al. 2016) and the photoperiod gene *PPD*-*D1*, respectively. The detection of these known effects confirmed the value of including this additional subset of highly significant but not-validated SNPs. The diagnostic marker for the *Vrn*-*A1a* allele (Yan et al. 2004) showed highly significant effects for HD (Table S7) in three environments and significant effects in a fourth environment (so is not included in Fig. S4). These *VRN1* diagnostic markers were not evaluated in the biparental populations so we do not know if *VRN*-*A1* would have been included in the validated SNPs.

Two additional sources of indirect evidence supported the validated SNPs. First, we found that several QTL for correlated traits were colocated and that their alleles were consistent with the sign of the correlations (Table 5). In addition, we found that several of the QTL identified in this study were in the same chromosome regions as QTL published in previous studies. This second criterion needs to be considered with caution because in some cases, there were no common markers and the comparison was based only on their relative positions on the chromosome (Fig. 3).

### QTL for grain yield and NWI3

We found a negative correlation between GY and NWI3 in all the environments tested in this study (Tables 2, 3), a result that is consistent with previous reports (Bowman et al. 2015). These highly significant correlations suggest that NWI3 could be a useful tool for indirect selection of yield in fall-sown spring wheats, a hypothesis that is also supported by the colocation or close linkage (< 1 cM) of six NWI3 and GY QTL. At these six loci, the parental alleles associated with higher water stress (higher NWI3) were also associated with decreased yield, which is consistent with the observed negative correlation. Since NWI3 and GY were measured at different times of the growth cycle and using independent methodologies, the colocation of these six QTL provides indirect evidence of their validity.

For grain yield, five of the 12 stable GY QTL identified in this study (Table 5) were close to previously mapped GY QTL (IWA2452, IWB38367, IWB62751, IWB35371, and IWB12366) and four were close to previous QTL for the correlated traits KW (IWB11049, IWB21128, and IWB38400) and KNS (IWB61210). These close QTL locations require further validation because Fig. 3 includes a large number (138) of previously published GY QTL.

For NWI3, six out of the 12 QTL identified in our study (Table 5) were closely linked with meta-QTL for drought stress detected by Acuña-Galindo et al. (2015) (Fig. S3). One additional NWI3 QTL on chromosome arm 5AL (IWA4813) was colocated with a QTL for lower leaf and spike temperatures detected under controlled conditions (Mason et al. 2011). These results provide additional support for the validity of the NWI3 QTL.

The frequency of favorable alleles for the validated GY and NWI3 QTL (Table S5) provides a useful tool to predict which QTL will have a positive impact in the largest number of lines within a given breeding program. We have prioritized the favorable allele for grain yield and yield components that are present at low frequency in the UCD breeding program (Table S5). However, since DuF and DuS accessions exhibit a different set of adaptive traits, additional experiments would be necessary to determine if the positive alleles identified here are useful in the spring-sown regions. For example, the favorable alleles for four GY QTL and three KW QTL detected here were at very low frequencies in the SDK and UMN breeding programs (Table S6). However, we do not know if this is because they were never introgressed in these programs or because they have different effects in the spring-sown environments.

### QTL for grain yield components

#### Kernel weight

This trait was positively correlated with grain yield and height and negatively correlated with spikelet number and heading time in all the environments tested in this study, and these correlations were significant in most environments (Table 3). Consistent with these correlations, five KW QTL were colocated with QTL for these traits (Table 5). In addition, eight out of our 13 KW QTL were closely linked with previously published QTL for KW on chromosomes 2A, 2B, 5A(2), 5B, 6A(2) and 6D (Fig. 3), providing indirect support to the validity of the identified KW QTL.

Kernel weight QTL 6A_IWB47942_ and 6D_IWB39422_ were closely linked to the *Gw2* gene (Fig. 3), a homolog of the RING-type E3 ubiquitin ligase that functions as a negative regulator of grain size in rice (Song et al. 2007). Induced mutations in this gene were confirmed to be associated with increased grain size in wheat (Simmonds et al. 2016). However, our study did not validate an SNP for KW in the cloned gene *TaGS5* reported before by Ma et al. (2016). Although this SNP is the same as IWB64668 segregating in our association panel, we did not detect significant effects for KW or other correlated traits.

#### Number of spikelets and kernels per spike

We validated 17 QTL for SNS but only one for KNS (Table 5, Fig. 3), which is likely associated with to the higher heritability of SNS relative to KNS (Table 4). A possible explanation for these differences is that SNS is determined early in the reproductive development (when the terminal spikelet is formed), whereas both yield and KNS are affected by environmental factors throughout the growing season. For example, environmental conditions that result in seed abortion or shattering after maturity would affect GY and KNS but not SNS. This hypothesis can also explain the higher correlation detected between KNS and GY than between SNS and GY (Table 3).

The strongest QTL for SNS detected in this study was associated with SNP IWA5912 on the long arm of chromosome 7A (Table 5). Su et al. (2016) detected a major QTL for KW and kernel length closely associated with IWA5913 (57 bp apart from IWA5912) using a diversity panel of 200 US winter wheat accessions. Since SNS and KW are negatively correlated (Table 3), we cannot rule out the possibility that the two QTL are caused by variation in the same gene. Other studies have also reported QTL for SNS and KW in this region, suggesting the presence of gene(s) with major effects on these traits (Fig. 3). To initiate the positional cloning of this gene, we identified two F_5_ lines from the Berkut × RAC875 biparental population with residual heterozygosity at the 7AL QTL region. These plants were self-pollinated to generate heterogeneous inbred families (HIFs) with reduced genetic variability to facilitate the precise mapping of this QTL.

In summary, as more yield and yield component QTL are mapped in wheat, a more precise delimitation of the QTL regions will be required to determine if linked QTL are caused by the same or closely linked genes. Figure 3 provides a preliminary view of closely located QTL that require a more precise characterization. The reference sequence of the wheat genome will facilitate these comparisons by providing a common coordinate system for sequence-based markers used in different studies. To facilitate this process we have deposited all the significant SNPs detected in this study with their genomic coordinates in the T3/Wheat database (https://triticeaetoolbox.org/wheat/).

##### Author contribution statement

CZ, SAG, EB, JH and TH performed field experiments and measured different traits. TH optimized canopy spectral reflectance methodologies. JZ coordinated the experimental part of the project and was the main person responsible for data analyses. AHC supervised SAG and coordinated experiments with CIMMYT. EA genotyped biparental populations. JZ wrote the first manuscript. JD initiated and coordinated the project, contributed to data analyses, provided extensive revision of the manuscript and wrote the final version. All authors reviewed the manuscript and provided suggestions.

## Electronic supplementary material

Below is the link to the electronic supplementary material.

**File S1**. *P* values and validation status of SNPs that passed the primary selection criteria in the GWAS. (XLSX 1688 kb)

**File S2**. Summary of comparison with previous studies. (XLSX 107 kb)

Supplementary material 3 (PPTX 1541 kb)

Supplementary material 4 (XLSX 85 kb)
