# Supplementary material for: Identification and validation of QTL for grain yield and plant water status under contrasting water treatments in fall-sown spring wheats
Source: Theor Appl Genet. 2018 May 16;131(8):1741–59. doi: 10.1007/s00122-018-3111-9 (PMC6061171; doi:10.1007/s00122-018-3111-9)
Supplement: Supplementary file 3 — Supplementary material 3 (PPTX 1541 kb) [file 122_2018_3111_MOESM3_ESM.pptx]

## Slide 1
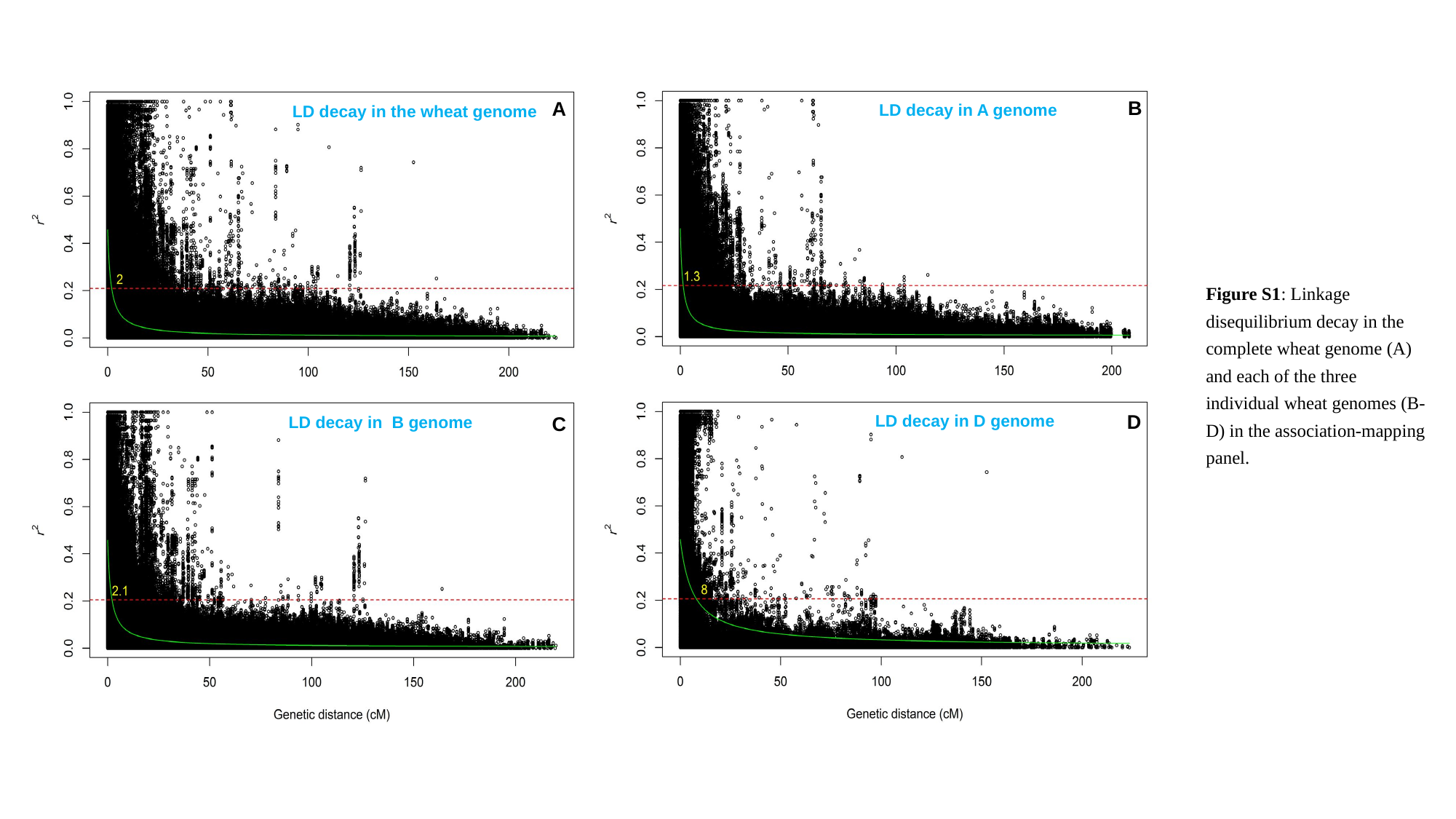

B
A
LD decay in A genome
LD decay in the wheat genome
D
LD decay in D genome
C
LD decay in B genome
Figure S1: Linkage disequilibrium decay in the complete wheat genome (A) and each of the three individual wheat genomes (B-D) in the association-mapping panel.

## Slide 2
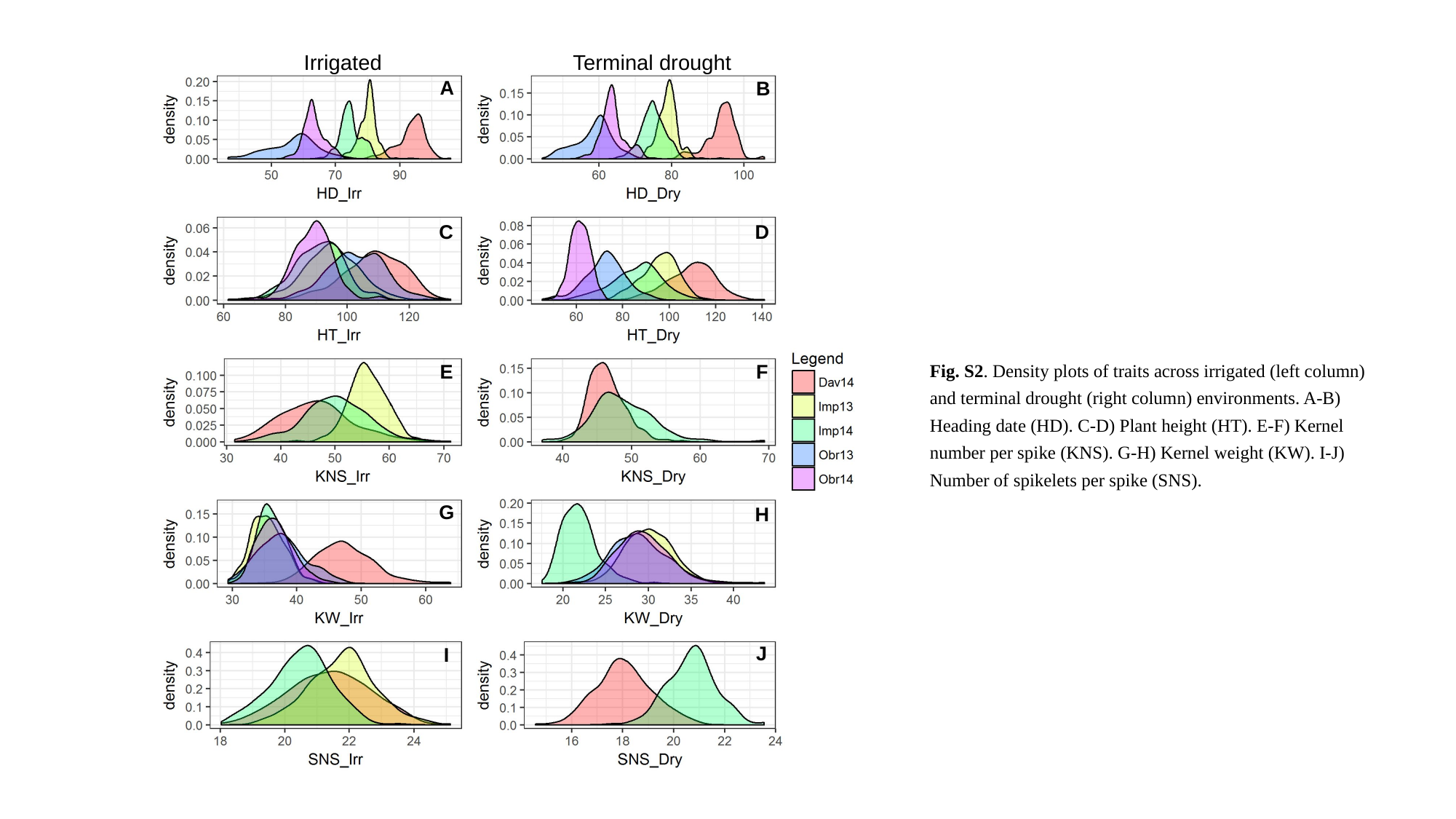

Irrigated Terminal drought
A
B
C
D
Fig. S2. Density plots of traits across irrigated (left column) and terminal drought (right column) environments. A-B) Heading date (HD). C-D) Plant height (HT). E-F) Kernel number per spike (KNS). G-H) Kernel weight (KW). I-J) Number of spikelets per spike (SNS).
E
F
G
H
J
I

## Slide 3
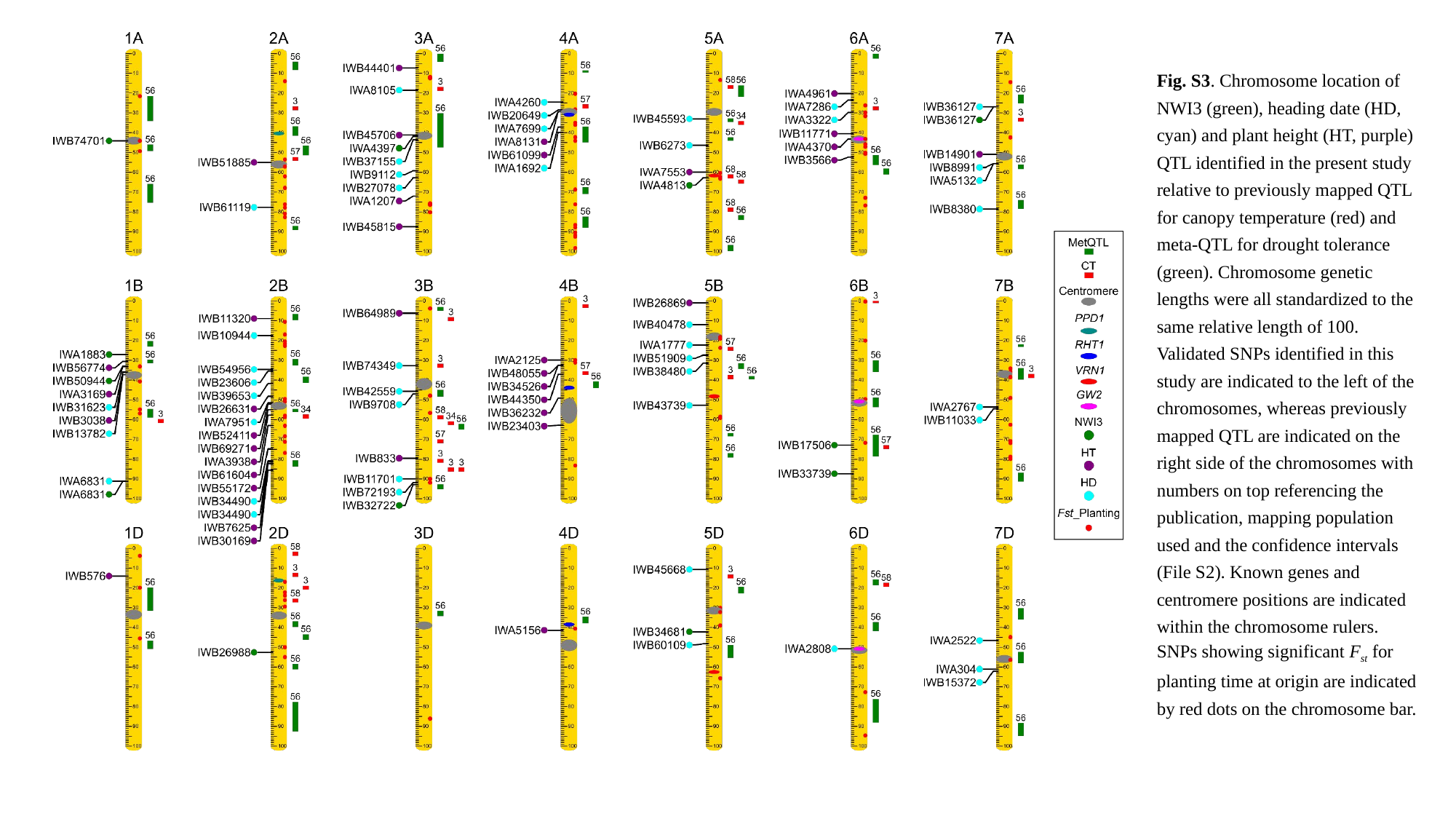

Fig. S3. Chromosome location of NWI3 (green), heading date (HD, cyan) and plant height (HT, purple) QTL identified in the present study relative to previously mapped QTL for canopy temperature (red) and meta-QTL for drought tolerance (green). Chromosome genetic lengths were all standardized to the same relative length of 100. Validated SNPs identified in this study are indicated to the left of the chromosomes, whereas previously mapped QTL are indicated on the right side of the chromosomes with numbers on top referencing the publication, mapping population used and the confidence intervals (File S2). Known genes and centromere positions are indicated within the chromosome rulers. SNPs showing significant Fst for planting time at origin are indicated by red dots on the chromosome bar.

## Slide 4
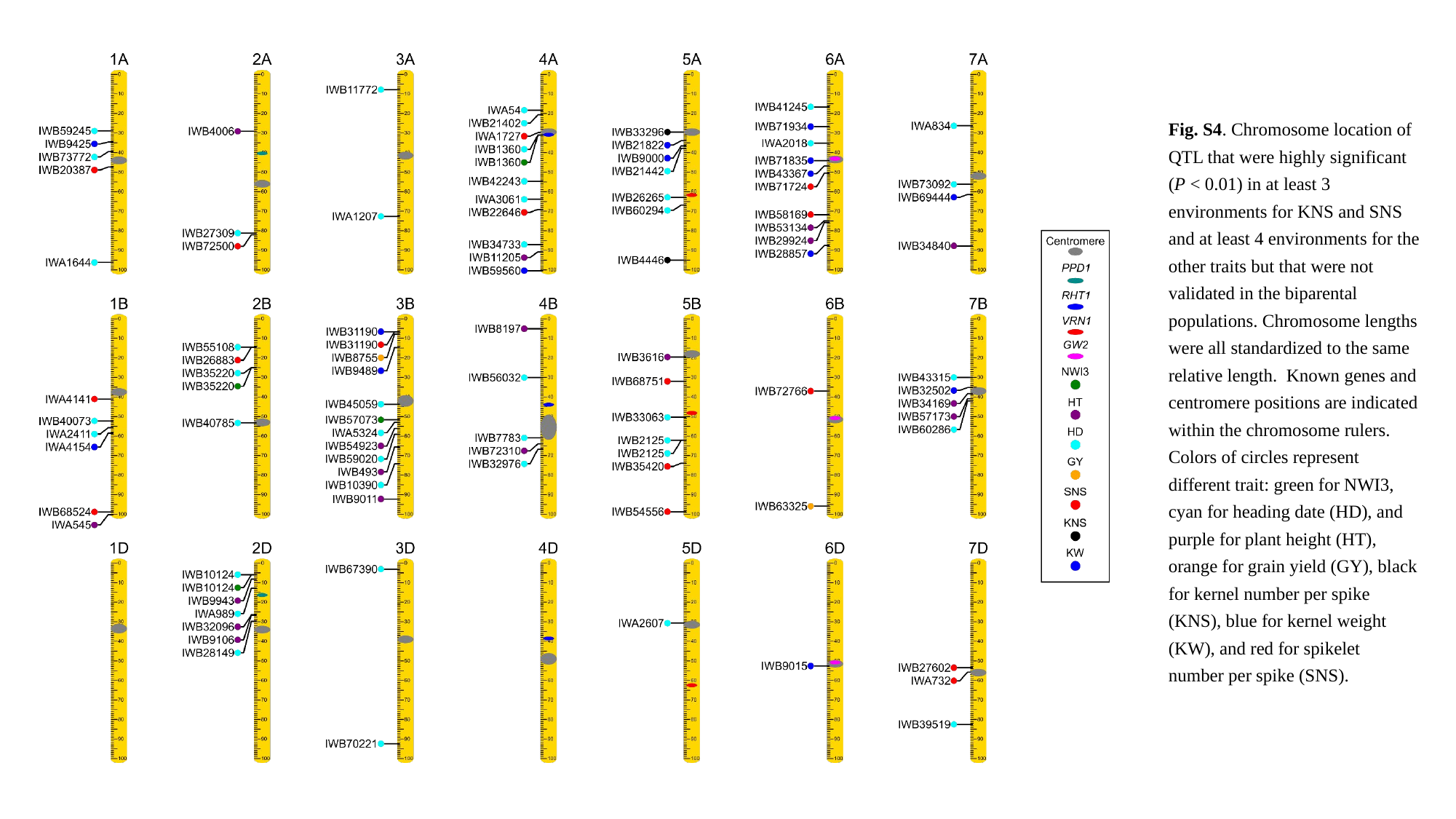

Fig. S4. Chromosome location of QTL that were highly significant (P < 0.01) in at least 3 environments for KNS and SNS and at least 4 environments for the other traits but that were not validated in the biparental populations. Chromosome lengths were all standardized to the same relative length. Known genes and centromere positions are indicated within the chromosome rulers. Colors of circles represent different trait: green for NWI3, cyan for heading date (HD), and purple for plant height (HT), orange for grain yield (GY), black for kernel number per spike (KNS), blue for kernel weight (KW), and red for spikelet number per spike (SNS).
